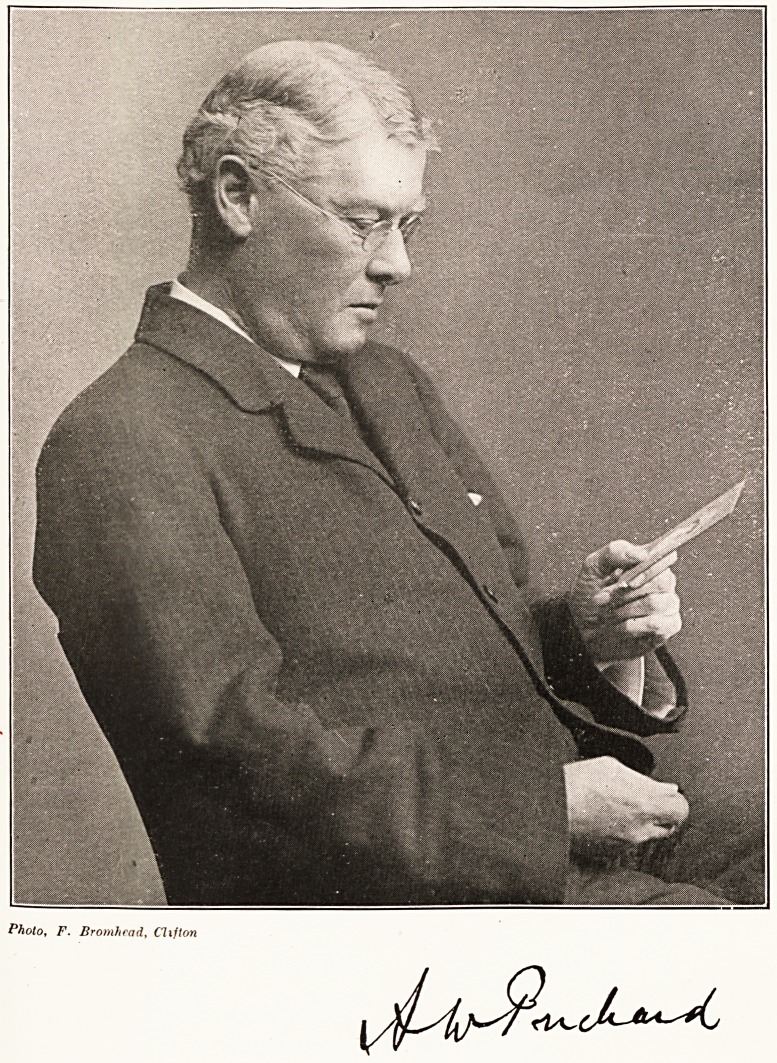# Arthur William Prichard

**Published:** 1926

**Authors:** 


### Obituary.

### ARTHUR WILLIAM PRICHARD, V.D., M.R.C.S.

With the death of A. W. Prichard, on January 13th, another old landmark of the medical profession in the West of England passed away. Born at the " Red Lodge," Park Row, Bristol, seventy-three years ago, he took a great deal of interest in this house, and during his life enriched its collection of old Bristol valuables by several gifts. He was the son of Augustin Prichard, a prominent surgeon on the staff of the Bristol Royal Infirmary, and grandson of Dr. James Cowles Prichard, F.R.S., the ethnologist, a physician of the Infirmary from 1816 for twenty-seven years, and so a Prichard—grandfather, son and grandson—has with only a brief interval been on the staff of this noble institution for considerably over a century.

A. W. Prichard was educated at Clifton College, where he entered early in 1863, commencing in the Junior School and finishing in the VI. Form before leaving in 1869. He then joined the old Medical School, and received his further medical training at the Infirmary and University College, London ; he qualified as M.R.C.S. in 1874, and shortly afterwards became House Surgeon to the Royal Western Ophthalmic Hospital, London.

In 1876 Prichard was appointed Honorary Assistant Surgeon to the Infirmary, and two years afterwards became full Surgeon, rising to Senior Surgeon and then to Consulting Surgeon in 1906, and was a member of the staff for fifty years. His was the last appointment under the old rule that physicians and surgeons were elected subject to the condition that they should not hold office for more than twenty years.

For considerably over fifty years he worked hard for the Bristol Eye Dispensary in Orchard Street. This Charity had been founded in 1812 by John Bishop Estlin. It is not too much to say that the institution would not have been kept going but for his great-nephew's energy in the professional work, and also for the keenness with which he obtained new subscribers when funds were required, particularly for the reconstruction and equipment of the old premises and the furnishing of up-to-date Out- and In-patient Departments worthy of its traditions. Prichard was also Honorary Consulting Oculist to the Royal School of Industry of the Blind.

A. W. Prichard was in his younger days an all-round athlete, gaining his cricket colours at Clifton College ; he afterwards played for Clifton, Thornbury and the Medicals. He was also a fair Rugby football player in the early days of the formidable Bristol Medical F.C. The writer of these notes remembers many an hour's cricket practice with Arthur Prichard in the garden of Chesterfield Place in the early seventies, when his father's windows suffered severely in consequence.

Over fifty years ago he was President of the Annual Medical Dinner, preceding such well-known medicos as Dr. J. Beddoe,

*Photo, F. Bromhead, Clifton*

A W Prichard

F.R.S., Dr. J. G. Swayne, and a host of others now long passed away. He was also President of the Bristol Medico-Chirurgical Society in 1895. An energetic volunteer officer, doing much good work with the Bristol Rifles (1st Volunteer Battalion Gloucestershire Regiment) until the Territorials were formed in 1908, when he became Officer Commanding the 3rd South Midland Field Ambulance, and also for some years Brigade Surgeon to the Portland Infantry Brigade.

When Augustin Prichard, his father, resigned the post of Surgeon to Clifton College he was appointed, and he held this post for many years. The Editor was a medical student under him, attended his lectures, and subsequently throughout the time he was Physician to Clifton College Arthur Prichard was his colleague there as well as at the Royal Infirmary, and his close friend.

He was on the staff of University College, Bristol, as Lecturer on Practical Surgery, and his pleasant manner and practical knowledge of the subject made these lectures interesting to the students. He was a member of the Ophthalmological Society, and made numerous contributions to the medical journals and societies during his long professional career.

Prichard was a quiet, well-read gentleman, always ready to lend a hand to anything that would promote the interest of the medical profession, and always very much against any idea of advertising himself; his cheery face and happy smile will be much missed by his numerous friends both old and young, and by a large circle of medical men in the West of England.

One of his hobbies was photography, and in this he excelled in the days when the amateur had largely to depend on his own initiative and experience both in taking and in developing his pictures.

The funeral service was held at the Clifton Parish Church, and was attended by a large number of his old friends and patients.

He leaves a widow, two sons, the elder being the Rev. Canon H. A. Prichard, of New York, the younger Lieut. N. Prichard in the Royal Navy, and three daughters, and fifteen grandchildren to mourn his loss. Few men have been more universally beloved than was Arthur Prichard.

## Figures and Tables

**Figure f1:**